# Sex education in adolescents with ASD: a qualitative study on the role of parents in the Italian context

**DOI:** 10.3389/fpsyt.2026.1712462

**Published:** 2026-03-23

**Authors:** Rosaria Ferrara, Pasquale Ricci, Alessia Succu, Ariana Calò, Monica Calderaro, Georgia Elisa Cafiero, Leonardo Iovino, Lidia Ricci

**Affiliations:** 1 Department of Anatomical, Histological, Legal Medicine and Orthopedic Sciences, Sapienza University of Rome, Rome, Italy; 2 Department of Life Science, Health and Health Profession, Link Campus University, Rome, Italy; 3 OISMA Organizzazione Italiana Studio e Monitoraggio Autismo, Roma, Italy; 4 Università degli Studi Internazionali di Roma, UNINT, Roma, Italy; 5 Department of Economic and Legal Studies, “Parthenope” University of Naples, Naples, Italy

**Keywords:** adolescent ASD, asperger, autism, sex health, sexual education

## Abstract

**Background:**

Sex education for adolescents with autism spectrum disorders (ASD) is often overlooked in traditional educational programs, despite scientific evidence highlighting the need for a specific personalized approach. Family involvement is essential, but Italian literature on the role of caregivers in this area is still limited.

**Materials and methods:**

This qualitative study adapted a previously developed qualitative protocol to the Italian context. A focus group was conducted with parents of adolescents with ASD (n=9) and an online questionnaire was administered (n=12). The questions explored previous experiences, perceptions of sex education, content considered a priority, and preferred methods of intervention.

**Results:**

The thematic analysis highlighted two macro areas: 1) the role of parents in sex education, ranging from direct supervision to delegation to professionals; 2) the vulnerability of children with ASD, linked to communication difficulties, exposure to inappropriate content and lack of experiential education. Parents expressed a strong need for clear, experiential and culturally sensitive educational tools.

**Conclusions:**

The study highlights the need to develop structured and inclusive sex education programs for adolescents with ASD, actively involving caregivers. A multidimensional and collaborative approach is essential, capable of integrating emotional, physical and regulatory aspects. The results offer a significant contribution to bridging the gap in Italy on this issue and to guiding future educational and policy interventions.

## Introduction

Autism Spectrum Disorder (ASD) is a complex, life-long neurodevelopmental condition with onset in early childhood, characterized by persistent differences in social communication and interaction, restricted and repetitive patterns of behavior, and atypical sensory processing (1, 2). ASD presents with marked clinical heterogeneity, both in symptom severity and functional outcomes, across individuals and developmental stages (3–5). These features can significantly affect multiple areas of functioning, including emotional regulation, interpersonal relationship, and social participation.

Beyond core diagnostic criteria, individuals with ASD often experience difficulties in understanding social cues, interpreting implicit or metaphorical language (e.g. irony), and managing emotional and relational exchanges (4, 6–10). Executive functioning differences, sensory sensitivities, and co-occurring neuropsychiatric condition (e.g. epilepsy, Tourette syndrome) may further complicate daily functioning and social adaption (3–5, 11–14). These characteristics have important implications for affective and sexual development, which is strongly intertwined with communication skills, emotional awareness, and social understanding.

For decades, autistic individuals have been stereotypically portrayed as asexual, emotionally immature, or uninterested in romantic and sexual relationships. Such representations have contributed to the marginalization of sexuality as a legitimate area of research and clinical attention in autism (15–17; T^18^). However, growing evidence over the past decades has challenged these assumptions, showing that autistic adolescents and adults experience sexual desire, romantic interest, and affective needs comparable to those of neurotypical individuals (19–21). Despite similar level of interest, autistic individuals often face grater difficulties in initiating and maintaining intimate relationship, as well as lower levels of satisfaction, due to challenges related to communication, emotional reciprocity, sensory processing, and understanding of social norms (22, 23).

Sexual education therefore represents a crucial component of well-being, autonomy, and personal safety for individuals with ASD. However, existing sex education programs are often poorly adapted to the specific cognitive, communicative, and experiential needs for autistic adolescents. Traditional approaches tend to prioritize biological aspects of sexuality while neglecting emotional education, consent, boundary recognition, and relational dynamics, which are particularly challenging areas for this population (18, 22).

The lack of adequate and accessible sex education increases the vulnerability of autistic adolescents to a range of risk, including misunderstanding social situations, exposure to inappropriate sexual content, and emotional or sexual victimization. Difficulties in recognizing consent, interpreting others’ intentions, and understanding social and legal boundaries may also lead to problematic behaviors, particularly in the absence of explicit and structured educational guidance (15, 22, 24, 25). These findings underscore the need for a comprehensive and developmentally appropriate approach to sex education that integrates biological, emotional, relation, and social dimensions.

Within this framework, family play a central role in the affective and sexual education of adolescents with ASD. Parents are often the primary source of information, emotional support, and behavioral guidance, especially in contexts where school-based sex education is limited or insufficient. At the same time, several studies have shown that caregivers frequently report feeling of uncertainty, embarrassment, and lack of adequate knowledge or tools to address sexual topics with their autistic children (21, 24). Communication difficulties, parental fears regarding vulnerability and safety, and the persistence of cultural taboos surrounding sexuality may further hinder open dialogue within the family context.

The parental role is particularly relevant during adolescence, a developmental phase characterized by profound physical, emotional, and relational changes. For autistic individuals, difficulties in emotional regulation and interpersonal functioning my persist across developmental stages and influence the experience of these transitions (11). Parents may experience ambivalent feelings, oscillating between the desire to protect their children and the difficulty of supporting their emerging affective and sexual autonomy. In some cases, this ambivalence can lead to avoidance or minimization of sexual topics, further limiting adolescents’ access to reliable and developmentally appropriate information (26).

Despite growing international attention to sexuality and autism, studies specifically exploring parental perspective on sex education for adolescents with ASD remain limited, particularly within the Italian context.

To address this gap, the present qualitative study explores parents’ perceptions of sex education for adolescents with ASD in Italy. By adapting the protocol developed by Lehan Mackin et al. (26) to the Italian context, this study aims to investigate caregivers’ experiences, concerns, perceived educational need, and preferred strategies for addressing sexuality with their children. Understanding parental perspectives is essential for the development of culturally sensitive, inclusive, and family-centered sex education programs that support the emotional well-being, autonomy, and safety of autistic adolescents.

## Materials and methods

The present study adopted a qualitative-dominant mixed methods design, in which qualitative data constituted the primary source of analysis, while quantitative data played a complementary and supportive role.

According to Creswell and Clark, qualitative-dominant mixed methods designs are particularly appropriate when the primary aim is to explore in depth participants’ experiences and meanings, while additional quantitative data are used to contextualize and enrich qualitative findings rather than to test hypotheses or generate statistically generalizable results (27).

In the present study, qualitative data collected through focus group discussions and open-ended questionnaire responses were prioritized to explore parents’ perceptions, experiences, and concerns regarding sex education for adolescents with ASD. Quantitative data derived from closed-ended questionnaire items were used to describe response patterns and to support the interpretation of qualitative themes.

### Participants

Participants included parents and primary caregivers of adolescents with Autism Spectrum Disorder (ASD), aged 14 to 20 years. To be eligible for the study, parents had to be literate and free from chronic mental or physical illnesses. Inclusion criteria for their children required a confirmed diagnosis of ASD, the manifestation of behaviors perceived as sexual, and the absence of concomitant intellectual disabilities, physical disabilities, or other psychiatric disorders.

The recruited caregivers represented different backgrounds in terms of age, education, and family composition. Despite the specific inclusion criteria, the adolescents presented heterogeneous clinical profiles, particularly regarding supports needs and communication abilities. This variability allowed the study to capture a wide range of parental experiences, reflecting the inherent diversity of the autism spectrum. Detailed demographic data were collected to contextualize participant’s responses and support the interpretation of qualitative findings.

Participants were recruited using a non-probability, purposive sampling strategy, consistent with the exploratory aims of the study. In qualitative and qualitative-dominant mixed methods research, such sampling approaches are considered appropriate when the objective is to obtain in-depth, contextually grounded insight rather than statistically representative data (28).

The sample size was determined in accordance with qualitative research standards, where the aim is depth of understanding rather than statistical representativeness. Data collection continued until thematic saturation was reached, defined as the point at which no new themes or relevant information emerged from additional data (29). In the present study, saturation was achieved through the combination of a focus group (n = 9) and an online questionnaire (n = 12). The convergence and repetition of themes across both data sources indicated that the collected data were sufficient to comprehensively capture parents’ perceptions and experiences regarding sex education for adolescents with ASD. Similar sample sizes have been reported in previous qualitative studies addressing sensitive and complex topics in autism research (24, 26).

The study involved voluntary participation in focus groups and anonymous online questionnaires conducted within standard educational and research practices. All participants provided written informed consent prior to participation.

Data collection took place between February and March 2025 with the collaboration of OISMA (Italian Organization for the Study and Monitoring of Autism).

### Procedure

The data collection process followed a qualitative-dominant mixed methods design and involved the use of two primary tools: focus group discussions and an online questionnaire. In line with this approach, qualitative data were prioritized to enable an in-depth exploration of parental experiences and perceptions, while questionnaire data served a complementary role by providing a structured overview of educational priorities and perceived needs.

A focus group was organized and led by a moderator with clinical and educational expertise in ASD. The decision to utilize a focus group was driven by the objective to replicate the study conducted by Lehan Mackin et al. (26), and to foster an environment where participants could openly share perceptions through group interaction.

The session were conducted in a protected, confidential setting after obtaining written informed consent. To ensure accuracy for subsequent analysis, discussions were audio-recorded, transcribed in full, and anonymized. The interview guide was adapted from (26) and focused on four questions:

- As a parent of a child with ASD, what role do you see yourself playing in providing sexual education to your child?
- Can you tell me about the sexual education your child has already received or might receive in the future, and what are your thoughts on it?
- What sexuality-related topics do you think your child should be aware of? Are there any topics you consider inappropriate for them?
- As a parent, what would be most helpful to you in providing sexual education information to your child? What have you done so far that has worked well?

In parallel, an online questionnaire was administered to 12 caregivers. This tool was chosen to collect more structured data, facilitating a quantitative analysis of trends, and to provide a private context for those who might find it difficult to discuss sensitive topics, such as their children’s sexuality, in a group setting.

The questionnaire was organized into sequential sections, beginning with informed consent and privacy protocols to ensure anonymity. This was followed by a demographic section collecting personal data such as gender, age of both caregiver and child, educational background, ethnicity, and family composition. The subsequent part focused on the child’s clinical profile, specifically the ASD diagnosis and the levels of support required in educational and communication settings. Finally, the questionnaire presented a combination of closed-ended questions addressing topics covered and preferred educational formats, as well as open-ended questions designed to mirror the qualitative depth of the focus group discussions.

### Data analysis

The data collection process followed a qualitative-dominant mixed methods design and involved the use of two primary tools: focus group discussions and an online questionnaire. In line with this approach, qualitative data were prioritized to enable an in-depth exploration of parental experiences and perceptions, while questionnaire data served a complementary role by providing a structured overview of educational priorities and perceived needs by (29).

Analysis proceeded through an iterative process of familiarization, coding, and theme development. Coding was conducted manually by two researchers independently; codes and themes were subsequently compared and discussed until consensus was reached, ensuring intersubjective reliability.

The analysis moved beyond descriptive categorization to an interpretative level, focusing on parents’ emotional experiences, perceived vulnerabilities of adolescents with ASD, and sociocultural factors shaping sexual education within the Italian context. Representative quotations were selected to illustrate each theme and to preserve participants’ voices.

The online questionnaire complemented the focus group data by providing more structured information and facilitating the identification of broader response patterns. This format also offered a more private context for participants who may have experienced discomfort discussing sensitive topics related to their children’s sexuality in a group setting.

Closed-ended questionnaire items were analyzed using descriptive statistics to examine frequencies and trends in parents’ responses regarding educational priorities, perceived risks, and preferred intervention formats. Open-ended responses were analyzed qualitatively using the same thematic framework applied to the focus group data, enabling systematic integration across data sources.

Overall, the combined use of qualitative and quantitative data strengthened methodological triangulation and contributed to the credibility and internal validity of the findings.

## Results

The qualitative analysis identified two interconnected thematic areas: (1) parental roles and practices in sexual education, and (2) sexuality, vulnerability, and socio-cultural influences. These themes emerged consistently across focus group discussions and open-ended questionnaire responses.

### Parental roles and practices in sexual education

Parents described heterogeneous approaches to sexual education for their adolescents with ASD, reflecting differences in personal beliefs, perceived competence, and available resources. Some caregivers reported relying on external professionals, such as sex educators or clinicians, to support their children in understanding sexuality and managing sexual behaviors. Others emphasized their own role in providing emotional presence and reassurance, framing sexual development as a normal and acceptable part of adolescence.

One parent reported seeking professional support early in adolescence to address emerging sexual behaviors and provide clear guidance on appropriate contexts and boundaries:

“As for my son, I had to become aware of the issue early on, already in middle school, because we all know how adolescence works. What I did was turn to a sex educator, and it went well, he was able to teach him how to manage those impulses, when and where.”

In contrast, other parents highlighted a more informal and relational approach, focused on normalization and emotional containment rather than structured instruction:

“My role was simply to be present during his discovery. I just reassured him that what he had discovered was not something abnormal. I told him to live those moments with serenity.”

Masturbation emerged as a salient and sometimes challenging topic within parental narratives (**Table 1**). Some caregivers reported addressing it openly as part of sexual education, providing explicit guidance about privacy and appropriateness. Others expressed uncertainty or discomfort, particularly when communication with their child was limited. Difficulties in emotional expression, reduced verbal abilities, and limited shared language were frequently described as barriers to effective dialogue about sexuality.

**Table 1:** Topics considered important by parents for sex education in adolescents with autism spectrum disorder (ASD).

What topics do you think teenagers should learn about in sex education?	n	%
Abortion	5	42%
Anatomy and physiology	7	58%
Self-assertion and self-defence	6	50%
Public/private behaviour	10	83%
Personal boundaries	8	67%
Consequences of sex	9	75%
Birth control	11	92%
Pregnancy	9	75%
Language and communication	8	67%
Masturbation	9	75%
Pornography	4	33%
Prostitution	6	50%
Puberty	9	75%
Unspoken rules in social interactions	6	50%
Interpersonal relations	9	75%

Several parents also reported feeling unprepared to address sexual topics due to their own lack of prior sexual education, describing a sense of inadequacy and the absence of appropriate tools or guidance. These experiences were often accompanied by frustration and concern about their ability to meet their children’s emotional and educational needs.

### Sexuality, vulnerability, and sociocultural context

A second thematic area concerned parents’ perceptions of sexuality as closely intertwined with vulnerability and exposure to risk. Caregivers frequently expressed concerns about their children’s ability to navigate social and sexual contexts safely, particularly in relation to misunderstanding social cues, interpreting others’ intentions, and recognizing appropriate boundaries.

Exposure to sexually explicit material, especially pornography, was identified as a significant source of concern (**Table 2**). Parents described apprehension about the impact of such content on their children’s understanding of sexuality and relationships, particularly in peer contexts where explicit material may be shared informally:

**Table 2:** Main parental concerns regarding sex education for adolescents with autism spectrum disorder (ASD).

What are your main concerns about sex education?	n	%
Lack of information or adequate resources	6	50%
Risk of exploitation or abuse	6	50%
Possible misunderstandings or inappropriate behaviour related to sexuality	7	58%
Expression of sexuality inappropriate to the person's level of development	3	25%
Sexual interests considered peculiar or atypical	2	17%
Exposure to pornography	5	42%
Contemporary issues related to sexuality	1	8%
Risk of social stigma	2	17%

“My only real concern is about exposure to the wrong kind of material. Being in a technical class with all boys, I see that he’s a bit disturbed by all the explicit videos shared around.”

Parents emphasized that sexual education for adolescents with ASD should extend beyond biological information to include emotional education, relational skills, and explicit teaching about consent, boundaries, and respect for others. Particular importance was attributed to supporting adolescents in understanding relationship dynamics, managing rejection, and developing body awareness.

Preferences regarding educational formats emerged from questionnaire data, with most parents expressing a desire for individualized educational plans tailored to the specific needs and characteristics of autistic adolescents (**Table 3**). Group-based interventions promoting peer interaction were also viewed positively, particularly when facilitated by professionals external to the family.

**Table 3:** Parental preferences regarding the most effective formats for sex education for adolescents with autism spectrum disorder (ASD).

Which format do you consider most effective for sex education?	n	%
Visual aids (e.g. images, videos, graphs to facilitate understanding)	5	42%
Interactive method (e.g. practical activities, discussions, quizzes)	4	33%
Adapted for people with autism spectrum disorder (ASD)	8	67%
Use of technology (e.g. online platforms, educational apps, e-learning)	1	8%
Repetition of content (to reinforce learning)	2	17%
Written material (clear and accessible texts for independent reading)	2	17%
Contextualised content (practical examples applicable to real life)	5	42%
Parental involvement (opportunity to give feedback and participate)	4	33%
Access to additional resources (links to further reading)	1	8%
Independent learning (structured to be followed at one's own pace)	2	17%
Diversification of methods (information presented in multiple formats to suit different learning needs)	0	0%
Practical examples of behaviour (concrete demonstrations of appropriate interactions)	5	42%
Behavioural therapy principles (strategies to improve understanding and social adaptation)	5	42%
Respect for personal and family values	4	33%
Group lessons (learning in a collective context)	3	25%
Affordable (resources available without financial barriers)	2	17%
Applicable at school (adaptable to the school context)	5	42%
Short duration (can be completed in a short time)	2	17%
Parental involvement (opportunity to address issues together with the family)	4	33%

Across narratives, sexuality was described as embedded within broader sociocultural influences, including norms, taboos, and limited public discourse around disability and sexuality. Parents highlighted the need for educational approaches that support adolescents with ASD in experiencing their sexuality in a conscious, informed, and safe manner, while also addressing their heightened vulnerability within social environments.

## Discussion

The findings of the present study highlight the complexity and heterogeneity of the parental role in sex education for adolescents with autism spectrum disorder (ASD). Consistent with the previous international research (19, 26), parents reported a wide range of experiences and educational approaches, ranging from proactive involvement to feelings of uncertainty and perceived inadequacy in addressing affective and sexual topics. This variability suggests the absence of structured and shared educational framework capable of supporting families in a consistent and equitable manner.

A central issue emerging from the qualitative analysis concerns the difficulty parents experience in addressing the emotional and sexual sphere, particularly in relation to communication barriers commonly associated with ASD (21). Many caregivers reported struggling to interpret their children’s emotional states or to respond effectively to sexual curiosity, often linking this difficulty to their own lack of prior sexual education and limited access to appropriate educational resources. These challenges appear to be particularly pronounced in adolescents with reduced verbal abilities or complex communication needs, reinforcing the need for sexuality education approaches that are specifically adapted to neurodivergent profiles.

In line with the findings reported by (18), parents demonstrated a strong awareness of their children’s socio-relational vulnerability. Difficulties in understanding social cues, implicit rules, and interpersonal boundaries were frequently described as factors increasing exposure to potential risks, including inappropriate content and unsafe interactions. Concerns regarding exposure to pornography and the lack of tools to interpret sexual norms and boundaries emerged as prominent themes across both focus group discussions and questionnaire responses. These parental perceptions are consistent with previous evidence highlighting increased vulnerability to adverse or potentially traumatic experiences among individuals with ASD compared to neurotypical peers (22, 30).

Importantly, parents emphasized the need for comprehensive sex education that extends beyond biological information to include emotional awareness, identity development, consent, relational skills, and the recognition of personal and interpersonal boundaries. Preferences expressed by caregivers favored experiential and structured educational interventions, often delivered by external professionals and implemented in small-group settings to facilitate peer learning, as also suggested by Calabrò et al. (15). Questionnaire data further supported these qualitative findings, indicating a strong demand for individualized and accessible educational pathways tailored to adolescents’ cognitive and communicative profiles.

The results also point to the relevance of the sociocultural context in shaping parental experiences and educational practices. Compared to the original study conducted in Iowa by Mackin et al. (16), the present findings suggest that within the Italian context the family assumes an even more central role in mediating affective and sexual education. Many parents reported having received little or no formal sex education themselves, which appeared to exacerbate feelings of unpreparedness and to widen the intergenerational gap in the transmission of affective and relational knowledge. This cultural dimension may act as a moderating factor influencing parental attitudes, perceived responsibilities, and reliance on external professional support.

Taken together, these findings underscore the importance of developing structured, culturally sensitive, and family-centered sex education programs for adolescents with ASD. A qualitative-dominant mixed methods approach allowed for an in-depth exploration of parental experiences while contextualizing these insights within broader educational priorities, highlighting convergent patterns across data sources. Future research adopting comparative and cross-cultural perspectives would be valuable in examining how different sociocultural frameworks, educational systems, and family roles influence parental approaches to sexuality education, thereby extending the relevance of these findings beyond the Italian context.

### Limitation

Several limitations of the present study should be acknowledged. First, the use of a small, non-probability sample and a purposive recruitment strategy limits the transferability of the findings beyond the specific sociocultural and institutional context in which the study was conducted. Although this approach is consistent with the qualitative-dominant mixed methods design and the exploratory aims of the study, caution is warranted when extending the results to other populations or settings.

Second, the study focused exclusively on parental perspectives. While caregivers play a central role in affective and sexual education, their accounts may reflect protective attitudes, interpretative frameworks, or difficulties in fully capturing the complexity of adolescents’ affective and sexual experiences. Future research may therefore benefit from adopting a multi-informant approach, including the perspectives of educators, clinicians, and other professionals involved in supporting adolescents with ASD, in order to provide a more comprehensive understanding of educational needs and practices.

A further limitation concerns the selective inclusion criteria, which focused on adolescents with ASD without intellectual disability or major comorbid psychiatric conditions. As a result, the findings cannot be generalized to the full autism spectrum, particularly to individuals with higher support needs, for whom affective-sexual education may present additional challenges.

A further limitation concerns the selective inclusion criteria, which focused on adolescents with ASD without intellectual disability or major comorbid psychiatric conditions. As a result, the findings cannot be generalized to the full autism spectrum, particularly to individuals with higher support needs, for whom affective-sexual education may present additional challenges.

In light of these limitations, future research would benefit from longitudinal designs aimed at monitoring the evolution of affective and sexual education needs across developmental stages, as well as the long-term impact of specific educational interventions. Additionally, comparative and cross-cultural studies are strongly warranted to examine how sociocultural norms, educational systems, and family roles shape parental approaches to sexuality education for adolescents with ASD. Such perspectives would enhance the global relevance of the findings and support the development of culturally sensitive and internationally applicable educational frameworks.

## Data Availability

The original contributions presented in the study are included in the article/Supplementary Material. Further inquiries can be directed to the corresponding author.
